# Erasmus Mundus Master of Bioethics: a case for an effective model for international bioethics education

**DOI:** 10.1007/s11019-017-9814-x

**Published:** 2017-11-10

**Authors:** Jan Piasecki, Kevin Dirksen, Hamilton Inbadas

**Affiliations:** 1 Department Philosophy and Bioethics, Faculty of Health Sciences, Jagiellonian University, Medical College, Michalowskiego 12, 31-126 Krakow, Poland; 2 Providence Center for Health Care Ethics, Providence Health and Services, 9205 SW Barnes Road, Portland, OR 97225 USA; 3 School of Interdisciplinary Studies, University of Glasgow, Crichton University Campus, Dumfries, Scotland DG1 4ZL UK

**Keywords:** Graduate education in bioethics, Erasmus Mundus Master of Bioethics, Bioethics education, Moral imperialism

## Abstract

Designing bioethics curriculum for international postgraduate students is a challenging task. There are at least two main questions, which have to be resolved in advance: (1) what is a purpose of a particular teaching program and (2) how to respectfully arrange a classroom for students coming from different cultural and professional backgrounds. In our paper we analyze the case of the Erasmus Mundus Master of Bioethics program and provide recommendations for international bioethics education. In our opinion teaching bioethics to postgraduate international students goes beyond curriculum. It means that such a program requires not only well-defined goals, including equipping students with necessary skills and knowledge, but also it should first and foremost facilitate positive group dynamics among students and enables them to engage in dialogue to learn from one another.

## Introduction

The nature and conditions of bioethics education considerably influences the quality of its clinical application and teaching for the student. An incompetent, ideological, or culturally insensitive bioethicist may have been a victim of poor bioethics education. Because this poorly educated bioethicist can serve as a policy maker, medical ethics teacher, member of an Institutional Review Board/Research Ethics Committee (IRB/REC), opinion leader, or at the bedside in the clinic, it is important to properly design and conduct bioethics education. In this paper we discuss a case of the Erasmus Mundus Master of Bioethics (EMMB)—a 1 year postgraduate academic program—and offer specific recommendations for international bioethics education.

In our short article, we not only analyze official documents and arguments in the debate on international bioethics education, but we also refer to our experience with the program as former EMMB students. Now 6 years removed from the aforementioned course of study, one of us JP is an assistant professor in Poland who teaches bioethics to medical students and does research on healthcare systems; another KD is a practicing bioethicist in a hospital system on the west coast of the United States; and the third one HI is a post-doctoral researcher studying end of life issues in the global context based in the UK. JP was also a visiting scholar of the last edition of the EMMB and participated in teaching activities in the course on research ethics.

In our opinion teaching bioethics goes beyond curriculum and the key for success is twofold: a well-designed curriculum and an educational infrastructure that is open to a dialogue with students and creating space for them to discuss and learn from one another. We are convinced that the EMMB met these two criteria for a high quality and effective bioethics education program.

International bioethics education encounters at least two critical issues. The first problem concerns a general goal of studying bioethics. This is especially important because students come from diverse cultural and professional backgrounds. A graduate degree in bioethics might provide students with a general overview of bioethical problems or train toward the development of requisite competencies in preparation for a certain role (Dudzinski et al. 2013). The second challenge is a possible charge of cultural or ethical imperialism. Some argue that international bioethics programs in Western countries not only impose foreign ethical categories, but point out a coincidence between launching international bioethics programs and suspicious clinical trials in developing countries (de Vries and Rott 2011; Chattopadhyay and De Vries 2008; Hellmann et al. 2015). International bioethics education can thus be seen as an instrument of potential subversion of developing nations.

In the forthcoming sections we will describe the EMMB program, next reflect on particular challenges of the EMMB and a charge of ethical imperialism. Finally we provide recommendations for international bioethics education.

## Erasmus Mundus Master of Bioethics

The EMMB program was operated in collaboration by three universities: University of Leuven, Belgium (KUL), Radboud University Nijmegen, the Netherlands (RUN), and University of Padua, Italy (PU). The program after ten editions came to its end in summer 2016 due to changes in financial support from European Commission and organizational transformations of collaborating universities. Since September 2016 KUL is continuing the Master of Bioethics program on its own.

The idea to create European program in bioethics emerged among collaborating European researchers in 1998 (KUL Website 2016). In 2001–2002 a prototype of the EMMB was launched in collaboration between KUL, RUN, UP and University of Madrid (KUL Website 2016). In its first edition it was a 2 year, part-time study program with a group of 15 students (Meulenbergs 2001). From the second edition the University of Madrid was substituted by the University of Basel. In September 2005 the Master of Bioethics was selected by the European Commission as an Erasmus Mundus Master course and was run in collaboration of the three aforementioned universities.

In the announcement published in *Medicine, Health Care and Philosophy* in 2007, one could learn that the EMMB is an international program, which nevertheless gives a special attention to European philosophical and theological traditions (Leysen 2007). The announcement contrasts European approach in bioethics with a dominant, but narrow Anglo-Saxon analytical tradition. The EMMB was established with an interdisciplinary character and it was focused on ethical problems arising in clinical situations (Leysen 2007). The curriculum evolved over the years, but it covered several important problems, theories, and methods discussed currently in international bioethical journals (see Table 1).

**Table 1:** A list of courses in 2010–2011 edition of the Erasmus Mundus Master

University	Title of the course	Short description of contents
KUL	Ethical Theories and Methods of Ethics	Lectures and discussion on diverse subjects: from care ethics, through personalism, deontology to utilitarianism. Ideas of Emmanuel Levinas, Jacques Lacan, Paul Ricoeur, Martin Heidegger, and Raimond Gaita were presented thoroughly
KUL/NU/UP	Interdisciplinary Research in Bioethics	Lectures, discussion and workshops on different methodological approaches to research in a field of bioethics. Students were supposed to present ethical analysis of a case, a literary review relevant to their research project, as well as present and discuss their research plans
KUL	Ethics of Reproductive Technologies	Lectures, discussion on several concepts and ethical problems concerning reproductive choices: responsible parenthood, screening for genetic diseases, childwish, reproductive cloning, religion and reproduction, gamete donation, stem cell research etcetera. A field trip to the Leuven University Fertility Center
KUL	Choices in Healthcare	Lectures, discussion, movies on the concepts of justice, rationing, access to healthcare and organ allocation
NU	Introduction to Bioethics	Lectures, discussion and workshops on the variety of subjects concerning philosophy of medicine, for instance: integrity of human body, the concept of personhood, literature and medicine, religion and bioethics
NU	Treatment Decisions for Vulnerability Populations	Lectures and discussion on several subjects surrounding the concept of vulnerability, for example: treatment decision in psychiatry, healthcare and undocumented immigrants, aging and healthcare, research with vulnerable populations
NU	Suffering, Death and Palliative Care	Lectures, discussion, workshops and movies on ethical aspects end-of-life decisions: palliative care, euthanasia, spiritual care, terminal sedation. A field trip to the nursing home
NU	Human Genetics and Medical Technology	Lectures, discussion, workshops and movies on ethical problems of new technologies in medicine: nanotechnology, stem cell therapy, enhancement, germline modification, genetic screening and testing, tissue engineering
UP	Clinical Bioethics	Lectures, discussion and workshop on clinical bioethics. Special attention was given to the concept of human dignity and establishing and operating of Healthcare Ethics Committee and Clinical Ethics Consultations. Field trips to Pediatric Department of Padova General Hospital and to field trip to General Hospital of Vicenza
UP	Religion and Bioethics	Lectures and workshops on bioethical issues in religious perspective. A general role of religion in culture and medicine was supplemented by the presentations on bioethics in Hinduism, Buddhism, Islam and Christianity
UP	Public Health and Prevention	Lectures, discussion and workshops on public health ethics and healthcare systems. A variety of problems concerning resources allocation, health inequalities in global context were discussed. A field trip to Istituto Zooprofilattico delle Tre Venezie Legnaro
UP	Research Ethics	Lectures, discussion and workshops on research ethics in biomedicine. The course presented the basic methodological concepts of clinical research, ethical framework for clinical trials as well as prepare to be a part of Research Ethics Committee

An important role among these was an introduction into empirical bioethics, qualitative methods, and a systematic review of literature: areas that were emerging in bioethics scholarship at the time. The EMMB gave students the possibility to develop their research skills. As part of the course assessment to complete the program students were offered the choice to write a paper intended for publication (instead of a thesis): a yearlong project consisting of several phases and assignments (Dirksen and Schotsmans 2012; Piasecki 2011). Another feature of the program was involving students into practicing ethical decision-making by role-playing ethics committee deliberations and debates on ethically-challenging cases. The program included several field trips to health care and research institutions that facilitated encounter and engagement with patients, caregivers, clinicians, and researchers as well as the ethical issues they face on a daily basis.

The interdisciplinary field of bioethics requires the involvement of many different specialists from diverse backgrounds. The EMMB managed to realize this task, and among the teaching staff were specialists from many different medical disciplines as well as from a variety of humanities and social sciences.

The EMMB was a truly international program, even if European students were overrepresented. For its 10 editions of more than 250 graduates came from more than 70 countries: 113 (44%) students from Europe, 65 (26%) from Asia, 33 (13%) from Africa, 12 (5%) from Middle East, 1 (< 1%) from Oceania, 21 (8%) from North America, and 11 (4%) from South America (Borry 2016). The graduates had also diverse professional backgrounds: medicine (92 students), theology (32), philosophy (31), law (15), biology (18), nursing sciences (12), social sciences (9), pharmacology (7), psychology (5), biotechnology (4), public health (4), biochemistry (3), chemistry (2), anthropology (2), international relations (1), languages (1), biomedical (1), biostatistics (1) (Borry 2016).

## The overall goal of the program

### First challenge: too ambitious and too diverse educational goals

One could wonder if the goals of the EMMB program were feasible and well-defined. Analyzing teaching materials of the courses one can imply that the overall goal of the program was at least threefold: (I) to introduce students to European intellectual traditions (phenomenology, personalism, hermeneutics) as European philosophy was thought to counter both a narrow biomedical concept of human beings as well as Anglo-saxon approach to bioethics, associated with principlism, utilitarianism, and thinking about morality in legal terms; (II) to build competency in ethical deliberations as students were introduced to the methods of case analysis and ethics committee role-playing was an important part of teaching; and (III) to introduce students to research methods and to equip them with skills needed to conduct research in the field of bioethics.

#### Problem of feasibility 1

The problem of feasibility has two facets: logistic and theoretical. The logistic aspect arises because of three factors: limited time, diversity of goals, and varied cultural and professional backgrounds of students. For a person trained and practicing as a physician to learn the underpinnings of a speculative approach of European philosophy in 2 or even 4 weeks is a difficult task. The main danger is that a student would get a glimpse of many different issues, but may not develop real understanding of them or receive a vague and possibly caricatured account of an otherwise complicated theory (Lawlor 2007). For example, the philosophical thought of Emmanuel Levinas would be unfairly distilled to merely “ethics is about the face of the other:” perhaps a temptation with limited time for a thoroughgoing treatment. Similarly, a complicated matter of genetics might be oversimplified and distorted for students with a background in humanities.

#### Problem of feasibility 2

The second aspect of feasibility concerns the nature of biomedical culture. One can argue that although the goal to counter and undermine the prevailing biomedical concept of human beings is praiseworthy, it is unrealistic. Byron Good and his research team conducted a participant observation of first year medicine students at Harvard Medical School. Good and his team also interviewed first year students in-depth. Their conclusion was that because “medicine formulates the human body and disease in a culturally distinctive fashion”, studying medicine is in fact introduction to this specific *medical culture* (Good 1993). According to Good students are taught how to see medical objects in everyday reality. Therefore in a relationship with a patient, they do not perceive a person with a biography, but an object of medical intervention with a medical history. Good also refutes, a so-called “conventional criticism” of medicine (Good 1993). For Good, a conventional critic of medicine assumes that if medical education is improved with the addition of more human and social sciences modules to the curriculum, students also will learn how to discern a real person, beside an object of medicine. But according to Good this criticism does not recognize that the medicine is fundamentally materialistic and it has already a “soteriological” dimension (Good 1993). Good clarifies that medicine does not neglect the spiritual (“soteriological”) dimension of human beings, but puts it into medical categories. A corollary to these is that the number of hours of ethics and humanities teaching medical students attend does not actually matter, because in a clinical context only medical, reductionist, and materialistic language is understandable. Therefore one can argue that instead of aiming at changing or undermining biomedical culture, a bioethics curriculum should rather focus on certain procedures and guidelines that can be introduced into a clinic within a framework of existing medical culture.

#### Problem of partiality

Another problematic issue is the intellectual merit of the the general goal discussed earlier (I). First, one can question geographical and ethnic approach to philosophical traditions. While “Continental philosophy” and “Anglo-saxon philosophy” are useful labels in less formal contexts, it is doubtful that this dichotomy is fruitful in a philosophical analysis of a certain problem. Moreover a closer examination of the development of European and US bioethics reveals mutual inspirations, personal connections and exchanges of ideas (Schotsmans 2015). Second, one can say that declaration of such a goal is in fact a declaration of being biased. Why should we assume, at the point of departure, that phenomenology gives better intellectual tools than philosophy of language? One could argue it should be the task of students to decide which arguments are more compelling. Thirdly, one can wonder: why present only European philosophy? Moral traditions of Islam, Indian, and Chinese civilizations are rich and should not be neglected.

A graduate program in bioethics may take at least two different forms (Dudzinski et al. 2013): (1) a master of arts degree program designed to provide students with a strong theoretical background of bioethical questions, (2) a master of science designed to provide students a set of competencies that can be applied concretely, for instance, in a clinical consultation setting, research ethics, or research in bioethics. Generally speaking, a master of science degree program is aimed at those who need specific competences. For instance, a person who started to serve as an IRB/REC member is likely to choose the second type of study. On this basis, one can argue that a master of arts degree program has too many theoretical and practical problems, as reviewed earlier, whereas a master of science degree program can be designed more precisely and uncontroversially with : (I) well-defined and feasible goals, and (II) a coherent target group. Therefore, we argue that all graduate bioethics degree programs should have a very specific, well-defined curriculum.

#### Feasibility 1

A one year post-graduate program should not be considered as an elementary introduction to many subjects. It should rather be conceived as a developmental opportunity. Students who choose to study bioethics have already at least a bachelor degree and are usually highly motivated. A course should provide them with a basis for their further research, reflections and exploration. Moreover classes should be designed in such a way that students can share their knowledge and skills with one another. Those who are more fluent in philosophy may help beginners, and likewise physicians and nurses can share their knowledge and experiences with those, whose background is in humanities. This approach can be beneficial for both parties. The objection of oversimplification concerns rather classes of ethics taught to medical students, who are not really interested in the subject and have really little time to study abstract ethical theories.

#### Feasibility 2

Good’s argument can be understood in two different ways: radical and moderate. According to a radical interpretation medical culture is so powerful that there is no place for ethics within a clinic. Therefore no matter how many bioethicists and bioethical commissions and committees are in or around a ward it does not have any influence on medical practice But it seems not really plausible. It is true that eventually almost any bioethical consideration takes a shape of a certain medical intervention. But what counts is the consideration that influences the decision. Moreover, if bioethicists, psychologists, chaplains, and family is present in a ward the other dimensions of human existence are not completely neglected. Thus there is a role for bioethics in a clinic and in biomedical world and that role can be broader than shaping policies and guidelines. A moderate interpretation agrees that there are some non-medicalized spheres with in a clinic (e.g. a talk with therapists, space for family meeting), but it keeps the main line of Good’s argument maintaining that bioethics education cannot change physicians’ medical attitude towards patients. Although this interpretation seems to be more plausible, it assumes that medical culture is totally monolithic in its materialistic reductionism. But being a doctor does not consist only of making purely medical decision, it implies also adherence to certain professional standards that exceeds narrow biomedical world. Some of these standards are of an ethical nature, for instance a standard of informed consent. Therefore bioethics education may target these dimensions of being a physician and enhances ethical standards of their behavior.

#### Partiality

Unfortunately it is very difficult to defend the EMMB against this charge. Partiality—even openly declared—is a flaw, because ideally the subject matter should be always presented in an unbiased and objective way. One can try to defend partiality on the grounds that philosophy is not science. In science there is only one official scientific paradigm that sets the norms of scientificity; in philosophy, in contrast, there are a few of different styles of thinking that are officially taught at the faculty (Zalewski 2000). There are only few philosophers who are experts in more than one competing style of thought. It would be too demanding to request that every philosophical institution have specialists in all styles of philosophy. In that case it is better to declare a certain approach than to claim that everything is presented objectively by experts in every conceivable speciality. By the same vein, one can defend the limited place for various traditions in bioethics outside of the European context (see Table 1). To summarize, although ideally all issues should be presented in an objective way, because this is impossible due to certain objective constraints (e.g., limited time), the possible bias in the focus of inquiry should be disclosed.

### Recommendation 1 and 2: clearly define the goals of the program and avoid partiality or at least declare it

The EMMB was in many respects a “classic” master of arts degree program, but with the potential to transform itself into a competency-based system *a la* traditional master of science degree programs.

There are two reasons why clear educational goals are important. First, the goal determines the content of the curriculum. The master of arts curriculum would consist of additional material in philosophy, ethics, and anthropology as opposed to a master in research ethics. Second, a master of arts and master of sciences are two different graduate degree programs largely addressed to different groups of students. Generally, the former suits to dual-degree holders who pursue an academic career and want to broaden and deepen their knowledge and skills in bioethics; the latter is addressed to those who already work in healthcare setting and want to change or broaden the scope of their work [e.g., serve on a IRB/REC (Dudzinski et al. 2013)]. Therefore these two different graduate degree programs in bioethics serve two different goals: both of which are needed. Healthcare needs competent specialists in bioethics who can serve in clinical consultation services as well as lawyers, physicians, and philosophers who have deeper and broader understanding of bioethical problems. In our opinion the EMMB realized all three goals it set, and for the authors here, formed very important year in our professional carriers.

Every bioethics program should avoid partiality and promotion of a certain approach. This is not only because ideas and reason should speak for themselves, but also it is ineffective as we write below referring to Pelegrino. Of course ideal impartiality is impossible due to many different constraints, but ideals are *ex definitione* impossible, though they serve us as directions.

## Cultural diversity

### Second challenge: cultural imperialism or managing cultural pluralism

Raymond De Vries and Leslie Rott draw an analogy between teaching bioethics to students from developing countries and missionary work (de Vries and Rott 2011). They interviewed students of one edition of the EMMB during their period of study in Leuven, Belgium. In their paper one finds criticism of the EMMB program. De Vries and Rott expressed their worry that a noble intent to spread ethical standards could lead to unwitting harm. The international standards do not really fit to the local circumstances of developing countries; consequently, students were left confused and/or with non-applicable knowledge. De Vries and Rott also noticed that some students felt themselves not listened to and that the communication had mainly one direction: from teachers to students. But there are even more serious accusations, that bioethics, and in consequence bioethics education, is a tool of moral imperialism (Chattopadhyay and De Vries 2008) and that there is a coincidence between launching international bioethics programs and conducting clinical trials in developing countries (de Vries and Rott 2011). Therefore, the second challenge for the EMMB was to create space for an intercultural meeting and dialogue. According to De Vries and Rott, the EMMB failed here.

Were the faculty of the EMMB program covert imperialists? This charge can be understood in two different ways. First, bioethics is a tool of imperialist power. While it may appear innocent or even noble, bioethics is, in fact, a projection of power. Some would call it *soft power*, but as long as it is power, it is an instrument of coercion. One way to avoid this charge is to share the power. In practice, this entails inviting students, scholars, and government representatives from developing countries to take part in designing curricula and teaching courses. Sharing the power and involving parties from developing countries is a good idea. Unfortunately, however, it must face two problems. First, it takes the imperialism charge for granted whereas the criticism is fragile to notable objections as we supply below. Second, it may not satisfy a kind of radical anti-imperialist who may hold that the involvement of students and scholars from developing countries is only a case of “indigenization” (de Vries and Rott 2011). Namely, those students from developing countries are used as means of colonization (Hellmann et al. 2016). At the beginning they are indoctrinated in the West, then sent back to a developing country to spread the western ethics and facilitate penetration of ethically suspicious research activities sponsored by foreign governments.

The second interpretation of the imperialism charge is symbolic. Bioethics teachers are not imperialists themselves, but there is an analogy between the approach of imperialists and that of teachers. Both imperialists and bioethics teachers do not listen and they are strongly convinced that they have something precious to offer, whereas people from developing countries should just listen and enjoy the nice gifts. In order to avoid this charge we have to find a common ethical ground. It can be an ethical theory that is acceptable by all and can be an official ethical background of the study program. Some argue that human rights are commonly accepted and understood ethical standards (ten Have 2010; Annas 2005). These proposals are not unproblematic and have notable limitations. First, human rights are a legal instrument that can be useful in the context of individual claims against a government. But it is not necessarily the ideal instrument for cultural and ethical conflicts that are not addressed by existing, recognized rights claims. Second, human rights are often general and, even where established, must still be interpreted in a concrete situation. This being the case, a reference to human rights as the normative backdrop for bioethical consideration is often insufficient to resolve specific, contextual moral problems. Consider for instance a ban on wearing Islamic headscarves. Some argue that the ban on full face veil violates human rights law because it limits individual religious freedom (Amnesty International 2010). But the proponents of the ban also refer to human rights and gender equality (Marshall 2006). Thus a declaration of commonly accepted rules is not sufficient for achieving resolution in the form of actual consensus and understanding. It seems that cultural openness and understanding cannot be just written down in a curriculum, but has to be present in teachers’ attitude and behavior.

There is also a possibility to debunk directly the imperialism charge. Those, who make this accusation may not have adequately considered two important facts. First, the international students are not only mere passive receivers of information. They could have been a bit overwhelmed at the beginning of their studies when surveyed by De Vries and Rott summarized above. But de Vries and Rott interviewed students very early in the course of their studies before being exposed to the rest of the curriculum and coursework. After the period of study in Leuven, Belgium, students travelled to Nijmegen, Netherlands and to Padua, Italy. In consequence students could not evaluate the whole program while being interviewed. In our experience, the EMMB students were intellectually-independent individuals, professionals of many kinds, who learned greatly one from another. Second, the imperialist critique overlooks a fact that, in a democratic and pluralistic society, any kind of cultural, religious or even philosophical indoctrination cannot succeed. Edmund D. Pellegrino writes “the best protection against indoctrination by someone else’s ethical values is possession of the skill of critical ethical judgment. This is precisely what a good class in ethics should provide”(Pellegrino 1989). Therefore teaching critical and independent thinking is the best antidote for ethical imperialism.

Although this reply to imperialist critique has its merits, it nevertheless not entirely sufficient. It does not take into account the significance of profound cultural and ethical differences. It may be true that the charge of moral imperialism is exaggerated, but the cultural differences and cultural misunderstanding might significantly interfere into the educational process. Also the education itself is not a passive receipt of content. To illustrate in the context of medical education, Federic Hafferty distinguishes three kinds of curriculum: a formal, an informal and a hidden (Hafferty and Franks 1994; Hafferty 1998). The formal curriculum is written down in official documents. The informal manifest itself at the interpersonal level and refer to a teacher a role-model for a student. The hidden curriculum invokes the organization culture and it has to be deciphered from the policy making, resource allocation, evaluations, and institutional slang. The hidden organizational culture and informal factors may have impact on the content of curriculum and the way it is actually realized. This factors should also be taken into account.

### Recommendation 3: create an open educational environment and involve your students

We submit that it is very difficult if not impossible to create an ethnically and culturally neutral message, but what really matters is not the message itself, but the forum: where critical ideas and reflections are discussed by the students. The values of program should be clearly declared, but there should be space to contest them. Matti Häyry writes that European values should not be treated as a tool of ethical colonization, but they can be a point of departure to promote discussion on significant aspects of bioethical issues (Häyry 2003). If the students are given intellectual tools, they can become even more critical, independent, and immune to indoctrination (cf. Pellegrino 1989). In our opinion, the key is organizational culture and the informal aspects of the educational program such as the promotion of dialogue and exchange. Also it is a great idea to create educational programs in active cooperation with students and scholars from developing countries, who can contribute to the teaching.

We hold that overall message of the EMMB program was not that of moral imperialism, but rather dialogue within moral and cultural pluralism. As the EMMB students ourselves, we gained a very unique experience of studying and living with persons from all over the world, in our case: from Indonesia, the Philippines, Germany, Slovakia, Nigeria, Malawi, UK, to name a few. The students came from different countries, but they had also diverse worldviews and religious background: Christian, Muslim, and atheistic. Our colleagues mostly were already experienced professionals who practiced as physicians, nurses, public health specialists, or had backgrounds in philosophy and theology. Suffice it to say, the dialogue amongst the students was vigorous and intellectually-stimulating: always enriched by representations of diverse philosophical and cultural worldviews. The discussion did not always reached consensus, but offered students critical insights and skills to appreciate a given problem or dilemma from many different points of view. We discussed not only ethical principles and cultural values, but also we were exchanging insights into what may be morally required in a particular set of circumstances.

## Conclusions

We have described the EMMB program as a mixed-model between theoretical- and competency-based education. That allows us to draw the first conclusion: that international bioethics programs should set clear and feasible educational goals. These goals determine the content of curriculum and allow students to assess whether a bioethics program meets their expectations and needs. We have also analyzed the charge of cultural imperialism. We have refuted this charge as flawed, but recognize the importance of cultural factors in bioethics education. In our judgment, the EMMB created an open space for ethical and cultural discussion with and among students as well as promoted cultural sensitivity and respect. This brings us to the conclusion that success in teaching bioethics requires not only a well-designed curriculum, but also an open and culturally sensitive culture that enables students to exchange ideas and engage in dialogue regarding the most challenging of ethical dilemmas.
